# Single venom-based immunotherapy effectively protects patients with double positive tests to honey bee and *Vespula* venom

**DOI:** 10.1186/1710-1492-9-33

**Published:** 2013-09-02

**Authors:** Johanna Stoevesandt, Bernd Hofmann, Johannes Hain, Andreas Kerstan, Axel Trautmann

**Affiliations:** 1 Department of Dermatology and Allergy, University Hospital Würzburg, Würzburg, Germany; 2 Department of Medicine, University Hospital of Mannheim, Mannheim, Germany; 3 Institute of Mathematics, Department of Statistics, University of Würzburg, Würzburg, Germany

**Keywords:** Anaphylaxis, Double sensitization, Field sting, Honey bee, Hymenoptera venom, Immunotherapy, Relapse, Risk factor, Treatment failure, *Vespula*

## Abstract

**Background:**

Referring to individuals with reactivity to honey bee and *Vespula* venom in diagnostic tests, the umbrella terms “double sensitization” or “double positivity” cover patients with true clinical double allergy and those allergic to a single venom with asymptomatic sensitization to the other. There is no international consensus on whether immunotherapy regimens should generally include both venoms in double sensitized patients.

**Objective:**

We investigated the long-term outcome of single venom-based immunotherapy with regard to potential risk factors for treatment failure and specifically compared the risk of relapse in mono sensitized and double sensitized patients.

**Methods:**

Re-sting data were obtained from 635 patients who had completed at least 3 years of immunotherapy between 1988 and 2008. The adequate venom for immunotherapy was selected using an algorithm based on clinical details and the results of diagnostic tests.

**Results:**

Of 635 patients, 351 (55.3%) were double sensitized to both venoms. The overall re-exposure rate to Hymenoptera stings during and after immunotherapy was 62.4%; the relapse rate was 7.1% (6.0% in mono sensitized, 7.8% in double sensitized patients). Recurring anaphylaxis was statistically less severe than the index sting reaction (*P* = 0.004). Double sensitization was not significantly related to relapsing anaphylaxis (*P* = 0.56), but there was a tendency towards an increased risk of relapse in a subgroup of patients with equal reactivity to both venoms in diagnostic tests (*P* = 0.15).

**Conclusions:**

Single venom-based immunotherapy over 3 to 5 years effectively and long-lastingly protects the vast majority of both mono sensitized and double sensitized Hymenoptera venom allergic patients. Double venom immunotherapy is indicated in clinically double allergic patients reporting systemic reactions to stings of both Hymenoptera and in those with equal reactivity to both venoms in diagnostic tests who have not reliably identified the culprit stinging insect.

## Background

Implementation of venom immunotherapy (VIT) in a Hymenoptera venom allergic patient involves a number of individual decisions weighing aspects of treatment efficacy and optimal protection versus costs, feasibility, and patient convenience. International guidelines [1-3] provide elaborate instructions on dosing and duration of VIT, but there is no definite statement on which venoms to include for immunotherapy if sensitization to more than one venom is detectable.

Sensitization to both honey bee and *Vespula* venom is a common finding in up to 59% [4] of Northern European Hymenoptera venom allergic patients. The terms “double sensitization” or “double positivity”, however, refer to the results of diagnostic tests rather than to their clinical significance. In the past decades, substantial efforts have been made to differentiate between venom “cross-reactivity” due to partial sequence identity of protein allergens or cross-reactive carbohydrate determinants and “independent double sensitization” by means of serological [4-11] or cellular tests [12-14]. Recently, assays of species specific IgE to recombinant major allergens such as rApi m1 and rVes v5 and lately rVes v1 [4-6] became commercially available for diagnostic testing. “Independent” or major allergen-based double sensitization has been considered an indication for double VIT [4,6,10,13], and some experts recommend that VIT should be extended to *all* venoms for which test results are positive and patients might potentially react to [15]. Still, there is a lack of clinical studies investigating whether double sensitization – regardless of its pattern or origin – is objectively associated with an increased risk of future anaphylactic reactions to both Hymenoptera venoms and therefore necessitates double VIT, entailing a significant increase of health care costs and time and effort.

Our study retrospectively examines a large cohort of patients who were treated by single venom-based immunotherapy at a standard dose of 100 μg per injection for at least 3 years between 1988 and 2008. It was designed to evaluate the risk of relapsing Hymenoptera-sting induced allergic reactions during maintenance VIT and after its discontinuation, to analyze risk factors for treatment failure, and to specifically compare the risk of field sting-induced anaphylaxis in mono sensitized and double sensitized patients.

## Methods

### Patients

In this retrospective single centre observational cohort study covering a period of 20 years (1988 to 2008), 721 patients were screened for study inclusion criteria. Patients with proven honey bee or *Vespula* venom allergy were eligible for evaluation if single venom-based immunotherapy had been performed according to international guidelines [1-3] and discontinued after a minimum period of 3 years. Patients who had received VIT with both venoms, those who stopped treatment before 3 years duration, and those who did not present for follow-up visits were excluded from the study. All patient-related procedures were part of routine diagnostic practice; written informed consent was obtained for allergologic work-up and initiation of VIT.

### Collection of data

Patients fulfilling study inclusion criteria were identified from patient files. A detailed follow-up history using a standardized questionnaire was routinely taken on the occasion of control visits in our allergy clinic. Data collection included information on patient-specific data (age at the time of VIT initiation, sex, underlying atopy or asthma) and on Hymenoptera field stings before, during and after VIT (date, number of stings, culprit insect, symptoms and severity of anaphylaxis, self-administration of medication, emergency treatment). Results from allergy testing (intradermal skin test results, specific IgE, baseline serum tryptase) and information on the course of VIT (time-interval between index-sting and VIT initiation, date of VIT initiation and duration of VIT) were retrieved from the patients’ files. Patients reporting relapses after discontinuation of VIT were invited for retesting and advised to restart VIT.

### Grading of anaphylaxis

Severity of anaphylaxis was classified as mild, moderate or severe with respect to cutaneous, respiratory, cardiovascular, gastrointestinal, and neurological symptoms according to a modified version of the system proposed by Muraro [16] as has been described previously [17].

### Allergy testing

Intradermal and serological tests were performed before initiation and prior to cessation of VIT. Intradermal skin tests with 10-fold serial dilutions of honey bee and *Vespula* venom (ALK-Abelló, Wedel, Germany) were carried out according to international guidelines [18,19]. Intradermal skin test responses at endpoint concentrations of less than or equal to 1.0 μg/mL were considered “positive” when resulting in a minimum wheal of 5 mm. Venom-specific IgE levels were determined using an enzyme immunoassay technique (DPC Biermann, Bad Nauheim, Germany) before 1998, which was replaced by the ImmunoCAP™ method (Thermo Fisher Scientific, Schwerte, Germany). The results were categorized by semi-quantitative classes ranging from 0 to 6. Classes higher than or equal to 1 were considered “positive”. Additional diagnostic methods including determination of specific IgE to recombinant major venom allergens were not routinely available during the study period. Determination of the baseline serum tryptase was introduced in routine diagnostic practice in 2002 and was performed using ImmunoCAP Tryptase™ (Thermo Fisher Scientific) in a subgroup of patients with a history of severe sting-induced anaphylaxis. Tryptase levels exceeding 11.4 μg/L (95^th^ percentile of the general population) were considered as elevated.

### Choice of venom for VIT

**Figure 1:**
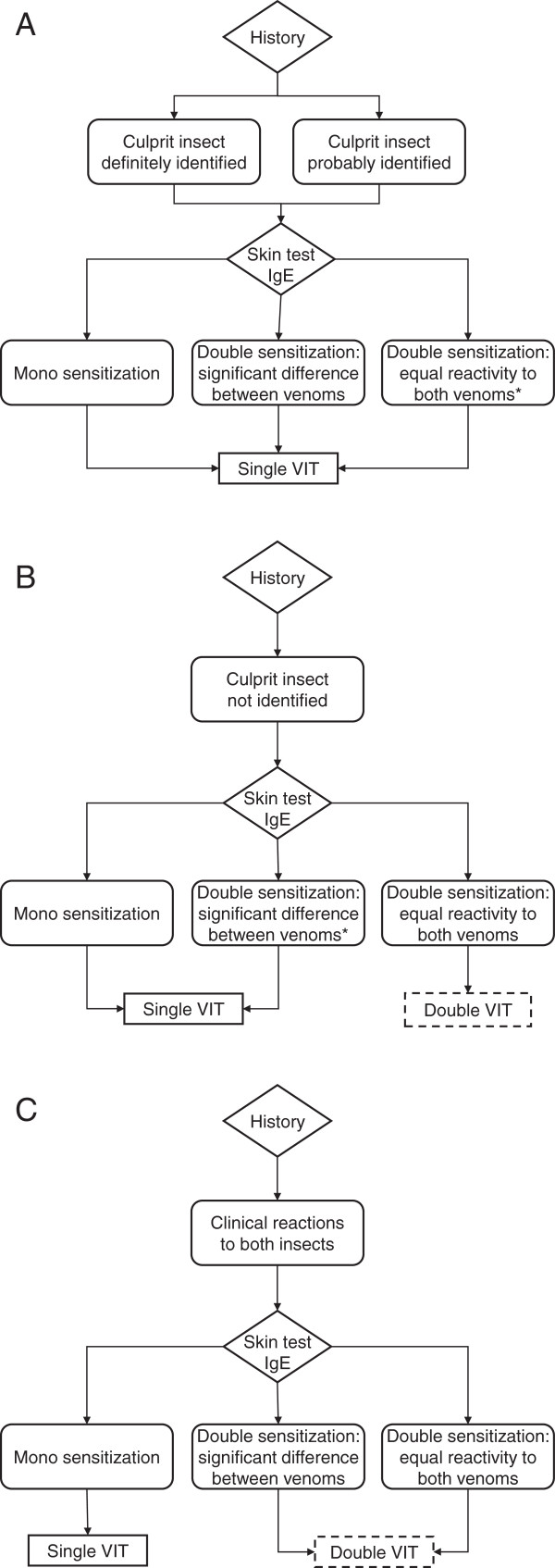
**Algorithm for the selection of venom for immunotherapy. A**. Reliable visual identification of the culprit stinging insect or sufficiently certain identification based on additional sting-related information (patient’s location and activity, time of the year, presence of a stinger left in the skin): administer single VIT. **B**. Culprit insect not identified: administer single VIT in mono sensitized patients or if sensitization to a single venom is significantly stronger than to the other (i.e. at least one serological class and one concentration step in intradermal tests). Administer double VIT in patients with equal reactivity to both venoms. **C**. Patients reporting anaphylaxis to stings of both insects: administer single VIT in mono sensitized patients. Administer double VIT in all patients with double sensitization.* Consider double VIT if additional risk factors are present (e.g. comorbidities, severe anaphylaxis to index sting, high degree of exposure). Dashed boxes: patients receiving double VIT were not eligible for study inclusion.

The venom for immunotherapy was selected based on a detailed clinical history and the results of skin and serological tests, taking into account the respective degree of reactivity to both venoms (Figure 1).

### VIT venoms and protocols

VIT was administered according to international guidelines [1-3] using standardized allergen extracts containing *Vespula* or honey bee venom. Reless™ (ALK-Abelló, Wedel, Germany) was used from 1988 to 1999, and ALK-lyophilisiert SQ™ and ALK-depot SQ™ (ALK-Abelló, Wedel, Germany) were used for build-up and maintenance phase since 2000. Before 2007, initiation of VIT was performed using a 5-day rush protocol which was subsequently replaced by a 3-day rush protocol. The maintenance dose of 100 μg was administered every 4 to 8 weeks according to the manufacturer’s instructions. Treatment was discontinued within 3 to 5 years in the majority of cases (96.5%); 22 patients (3.5%) pursued extended VIT for individual reasons.

### Definition of patient groups for statistical analysis

Patients were defined as “double sensitized” in the case of a positive intradermal skin test response and/or serological detection of specific IgE to both, honey bee and *Vespula* venom. A subgroup of 116 individuals comprised patients with equal reactivity to both venoms in diagnostic tests as well as a small number of patients (n=14) with discrepant diagnostic results (i.e. skin test reactivity was stronger to one and serum specific IgE to the other venom).

### Statistical analysis

Results are presented as numbers and frequencies for categorially scaled measures or as median and range for metrically scaled data. In case of metric data, the Wilcoxon signed-rank test was performed for the comparison of two dependent measures and the Mann–Whitney test for unpaired samples. To assess the relationship between two independent categorial measures, the chi-square test or the exact test of Fisher were used as appropriate. In case of dependent categorial data the Bower test was conducted. The binomial test was done for the comparison of two proportions. All tests were two-tailed and differences were considered statistically significant if *P* < 0.05. The software package used was IBM SPSS for Windows, version 19.0.

## Results

### Clinical data

The files of 721 patients with proven honey bee or *Vespula* venom allergy who had received VIT between 1988 and 2008 were screened for study inclusion criteria. Of 679 individuals who had completed at least 3 years of VIT, 14 patients who had received VIT with both venoms were excluded from evaluation. 30 patients were lost to follow-up, e.g. due to change of residence or death from causes unrelated to Hymenoptera venom allergy. Thus, complete data of 635 patients who had received single venom-based immunotherapy were available for evaluation as summarized in Table 1. The median age at the time of VIT initiation was 39 years (ranging from 5 to 77). The sex ratio was almost balanced with only a slight male predominance (50.7%). Of 635 venom allergic patients, 351 (55.3%) were double sensitized to honey bee and *Vespula* venom as detected by intradermal tests or serum specific IgE. The majority of double sensitive cases (n=160) were detected by both, serologic and intradermal tests (Table 2). 107 patients were double positive only serologically, and 84 only in skin tests. Double sensitization was more prevalent in individuals with honey bee venom allergy compared to those who had reacted to Vespula stings (77.9% versus 48.4%, P < 0.001). 135 (21.3%) patients had a history of severe (grade III) reactions following pre-VIT Hymenoptera field stings, 321 (50.6%) index sting reactions were moderate (grade II), and 171 (26.9%) were classified as mild (grade I). In 8 venom-sensitized patients (1.3%) with a history of large local sting reactions, VIT had been performed for individual reasons (mainly loss of quality of life due to fear of Hymenoptera stings). The mean duration of VIT was 3.9 years (ranging from 3 to 13) years. In the whole study population, no major side effects of VIT injections were reported. The mean observation time following discontinuation of VIT was 7.0 years (ranging from 1 month to 17 years).

**Table 1:** Clinical data of the cohort

	**n**	%*
Number of patients	635	
Median age at time of VIT-initiation (years) (range)	39	(5–77)
Male/female	322/313	
Results of allergy testing		
*Vespula* (*vulgaris*/*germanica*) mono sensitization	251	39.5
Honey bee (*Apis mellifera*) mono sensitization	33	5.2
Double sensitization	351	55.3
Severity of anaphylaxis to pre-VIT index field sting		
Large local reaction	8	1.3
Grade I (mild)	171	26.9
Grade II (moderate)	321	50.6
Grade III (severe)	135	21.3
Treatment course		
Mean duration of VIT (years) (range)	3.9	(3–13)
Mean observation time after VIT (years) (range)	7.0	(0–17)

* Percentages refer to the total study group of 635 patients.

**Table 2:** Mono sensitization and double sensitization detected by intradermal tests and serum specific IgE

**Serum specifc IgE**			
		**Mono sensitization**	**Double sensitization**
**Skin test**	**Mono sensitization**	284	107
	**Double sensitization**	84	160

### Skin test and serological reactivity before and by the end of VIT

Skin test reactivity and specific IgE significantly decreased during VIT (*P* < 0.001). By the end of treatment, intradermal tests with the venom used for immunotherapy were negative in 67 (10.6%) patients, and serum venom-specific IgE was negative (< 0.35 kU/L) in 128 (20.2%) patients.

### Risk of relapse and risk of severe reactions during and after VIT

396 of 635 patients reported at least one Hymenoptera sting after initiation of VIT by either honey bee (n = 74), *Vespula* (n = 233), European hornet (n = 2), bumble bee (n = 1) or by an unidentified stinging insect (n = 86), resulting in a re-sting rate of 62.4%. 174 (27.4%) patients got stung on several occasions: 98 (15.4%) patients had 2 stings, 34 (5.4%) patients had 3 stings, 20 (3.1%) had 4 stings, 8 (1.3%) had 5 stings, and 14 (2.2%) had more than 5 stings – several stings on the same event were counted as *one* sting. In case of repeated stings, details on the most severe or – if all stings were tolerated – the most recent field sting were documented: the outcome of 396 field stings (230 of which occurred in double sensitized patients) is summarized in Table 3. 150 field stings took place during VIT, 246 occurred after stopping treatment. Of 396 patients re-stung, 28 reported systemic reactions, implying an overall relapse rate of 7.1%. During VIT, systemic reactions occurred in 5 stings, and no severe reactions were reported. After discontinuation of VIT, 23 stings led to systemic reactions, 2 of which were classified as severe. Recurring anaphylactic reactions were found to be significantly less severe than the pre-VIT index sting reaction (*P* = 0.004). Characteristics of the 28 patients who suffered systemic anaphylaxis following Hymenoptera field stings during or after VIT are shown in Table 4.

**Table 3:** Clinical outcome of Hymenoptera field stings during and after VIT

	**No reaction**	**Anaphylactic reaction**			**Total**
		**I (mild)**	**II (moderate)**	**III (severe)**	
All stings	368 (92.9%)	13 (3.3%)	13 (3.3%)	2 (0.5%)	396 (100%)
Mono sensitized patients	156 (94.0%)	6 (3.6%)	4 (2.4%)	0 (0%)	166 (100%)
Double sensitized patients	212 (92.2%)	7 (3.0%)	9 (3.9%)	2 (0.9%)	230 (100%)
Stings during VIT	145 (96.7%)	3 (2.0%)	2 (1.3%)	0 (0%)	150 (100%)
Mono sensitized patients	67 (98.5%)	1 (1.5%)	0 (0%)	0 (0%)	68 (100%)
Double sensitized patients	78 (95.1%)	2 (2.4%)	2 (2.4%)	0 (0%)	82 (100%)
Stings after VIT discontinuation	223 (90.7%)	10 (4.1%)	11 (4.5%)	2 (0.8%)	246 (100%)
Mono sensitized patients	89 (90.8%)	5 (5.1%)	4 (4.1%)	0 (0%)	98 (100%)
Double sensitized patients	134 (90.5%)	5 (3.4%)	7 (4.7%)	2 (1.4%)	148 (100%)

**Table 4:** **Characteristics of 28 patients suffering re**-**sting reactions during or after VIT**

**Patient no.**	**Age**^**1**^**(y)**	**Sex(m/f)**	**BST(μg/L)**	**Allergy testing**								**VIT duration (y)**	**Post**-**VIT observation time (y)**	**No. of stings**^**4**^	**Field sting**^**5**^	**Severity of reactions (grade I-III)**		
				**Before VIT**				**End of VIT**										
				**IgE**^**2 **^**bee**	**IgE**^**2 **^**vesp**	**ID**^**3 **^**bee**	**ID**^**3 **^**vesp**	**IgE**^**2 **^**bee**	**IgE**^**2 **^**vesp**	**ID**^**3 **^**bee**	**ID**^**3 **^**vesp**					**Before VIT**	**During VIT**	**After VIT**
**Patients receiving honey bee VIT**																		
1	43	m	n.d.	3	0	n.d.	neg	3	0	0.1	neg	5	14	>5	bee	II	-	I
2	20	m	n.d.	6	1	0.001	1	2	0	0.1	neg	4	9	1	bee	II	LR	I
3	39	f	n.d.	3	1	0.01	neg	1	0	1	1	3	10	3	bee	II	LR	I
4	53	f	n.d.	2	0	0.001	1	2	0	0.01	0.1	3	14	1	bee	II	I	-
5	53	m	n.d.	6	4	0.001	0.01	3	0	0.001	0.1	5	8	1	vesp	III	-	I
6	37	f	n.d.	3	2	0.1	neg	2	1	0.1	neg	3	2	1	bee	II	-	I
7	26	m	n.d.	3	2	0.01	0.01	2	1	0.01	0.1	6	13	5	u	III	-	II
8	11	m	n.d.	3	4	0.001	0.1	3	3	0.1	0.01	5	2	4	u	II	II	LR
9	28	f	n.d.	4	2	0.001	0.01	2	0	0.1	0.1	5	12	2	bee	III	-	II
**Patients receiving *Vespula *VIT**																		
10	18	f	n.d.	0	3	neg	0.01	0	2	neg	0.01	5	12	2	vesp	II	LR	I
11	50	f	7.63	0	2	neg	0.1	0	3	neg	1	5	2	2	vesp	II	LR	II
12	39	f	n.d.	0	2	neg	0.1	0	0	neg	0.01	3	8	6	vesp	III	-	I
13	21	m	4,97	0	3	neg	0.001	0	2	neg	0.1	3	8	3	vesp	II	-	II
14	47	f	n.d.	0	2	neg	0.1	0	2	neg	1	5	4	1	vesp	III	-	II
15	51	m	n.d.	0	3	neg	0.001	0	0	neg	0.1	3	11	1	vesp	III	-	I
16	48	f	n.d.	0	2	neg	0.01	0	2	neg	0.1	5	4	1	u	I	-	II
17	30	f	n.d.	0	3	neg	0.1	0	0	neg	1	3	10	1	u	II	I	-
18	56	f	n.d.	0	5	neg	0.001	0	3	neg	0.1	5	7	1	vesp	II	-	I
19	32	m	n.d.	1	1	neg	0.1	0	0	1	0.1	4	13	3	vesp	III	LR	II
20	54	m	6.69	3	2	1	0.1	2	2	neg	0.1	3	13	4	vesp	III	-	III
21	53	m	110,0	3	3	0.1	0.01	0	1	1	1	3	11	3	u	III	-	II
22	45	m	n.d.	2	2	1	0.01	1	3	neg	0.1	5	4	3	vesp	III	II	-
23	30	m	n.d.	3	4	0.1	0.001	0	1	1	0.1	3	9	2	vesp	I	-	III
24	55	f	n.d.	2	2	0.01	0.01	0	0	1	0.1	4	8	2	vesp	II	I	LR
25	40	m	n.d.	2	3	0.01	0.001	n.d.	n.d.	n.d.	n.d.	3	14	1	u	III	-	I
26	37	m	n.d.	2	3	0.01	0.01	2	2	0.1	1	3	9	3	u	III	-	II
27	39	f	n.d.	2	5	1	0.001	1	2	1	0.1	6	9	2	vesp	II	-	II
28	46	f	n.d.	2	2	neg	n.d.	2	2	1	0.1	3	13	3	vesp	I	-	II

^1^Age at the time of VIT initiation, ^2^figures refer to semi-quantitative serological classes, ^3^figures refer to intradermal test endpoint concentrations (μg/mL), ^4^number of Hymenoptera stings after VIT initiation, ^5^Hymenoptera sting eliciting systemic reaction during or after VIT.

Abbreviations: *bee* honey bee, *BST* baseline serum tryptase, *ID* intradermal skin test, *LR* local reaction, *n.d.* not determined, *neg* negative, vesp, *Vespula* species, *VIT* venom immunotherapy, *u* unknown.

### Risk factors for recurring sting-induced anaphylaxis during or after VIT

Of several putative risk factors or predictors for systemic reactions to Hymenoptera field stings during or after VIT, only one reached statistical significance: severe anaphylaxis following the pre-VIT index sting was clearly related to future recurrence (*P* = 0.02). Tendencies towards an increased risk of relapse in patients reporting repetitive field stings after initiation of VIT (*P* = 0.07) or patients receiving honey bee VIT compared to those receiving *Vespula* VIT (*P* = 0.50) did not reach statistical significance. Patients with recurring Hymenoptera-venom induced anaphylaxis tended to be older than patients who tolerated field stings, but this was not statistically significant (*P* = 0.40). No relation could be established between recurrent anaphylaxis and the patient’s sex (*P* = 0.70), underlying atopy (*P* = 0.15) or asthma (*P* = 1.0), life-time number of Hymenoptera stings (*P* = 0.46), the decline of skin test reactivity (*P* = 0.45) or specific IgE (*P* = 0.12) during VIT, the time interval from index sting to initiation of VIT (*P* = 0.67), and the duration of VIT (*P* = 0.56).

### Treatment efficacy in mono sensitized and double sensitized patients

The overall relapse rates during and after VIT were 6.0% in mono sensitized and 7.8% in double sensitized patients. Though there was a tendency towards an increased risk of treatment failure, double sensitization was not significantly related to relapsing sting-induced anaphylaxis (*P* = 0.56, Table 5). This tendency, while not reaching statistical relevance, was more pronounced in the above defined subgroup of double sensitized patients with equal reactivity to both venoms in diagnostic tests (*P* = 0.15).

**Table 5:** Impact of double sensitization on the outcome of treatment

	**Mono sensitized patients**	**Double sensitized patients**		**Total cohort**
		**All**	Subgroup*	
Tolerated field sting	156 (94.0%)	212 (92.2%)	73 (89.0%)	368 (92.9%)
Relapse	10 (6.0%)	18 (7.8%)^1^	9 (11.0%)^2^	28 (7.1%)
Sum	166 (100%)	230 (100%)	82 (100%)	396 (100%)

*Subgroup of double sensitized patients comprising individuals with equal reactivity to both venoms in diagnostic tests.

^1^*P* = 0.56 (*P*-values result from statistical comparison of the risk of relapse in mono sensitized and double sensitized patients).

^2^*P* = 0.15.

## Discussion

### Standard dosed single venom-based immunotherapy over 3–5 years results in effective and long-lasting protection

VIT constitutes an effective treatment to protect venom allergic patients from sting-induced anaphylaxis [20]. Protection is highest during the maintenance phase and tends to decrease after discontinuation of treatment, resulting in overall relapse rates of approximately 10 to 15% [15,21-27]. Relapses following VIT-initiation tend to be less severe than pre-VIT sting reactions [23,24,27], but serious and even fatal Hymenoptera venom-induced anaphylaxis does recur on rare occasions [22,28-30]. We herein present the hitherto largest [22-26,31] single centre observational cohort study investigating the long-term outcome of VIT in a group of 635 Hymenoptera venom-allergic patients who received standard dosed treatment for 3 to 5 years between 1988 and 2008 (post-VIT observation time was up to 17 years). In contrast to other studies based on either challenge stings [27,32-35] or field-sting-related observations [23-26,31], all patients included received single VIT. Basic epidemiologic findings such as a median age of 39 years at the time of VIT initiation, a slight male preponderance being more pronounced in bee venom allergic patients [36], and a 21.3% rate of severe anaphylactic reactions following pre-VIT index stings as well as the re-exposure rate of 62.4% (to any Hymenoptera sting after VIT-initiation) and the overall relapse rate of 7.1% (that is 3.3% during VIT and 9.3% after its discontinuation) are roughly in line with those reported in earlier studies. Recurrent anaphylaxis to Hymenoptera field stings was significantly less severe than the pre-VIT index sting reaction (*P* = 0.004) which is again in accordance with previous findings [24,27]. Grade III anaphylaxis recurred in only 2 individuals (Table 4, patients 20 and 23) following discontinuation of treatment (that is 0.5% of 396 patients suffering re-stings). Number 23 was the only patient to develop more severe anaphylaxis on the occasion of his post-VIT field sting than before VIT. He got re-stung 6 years after discontinuation of treatment by more than 20 yellow jackets emerging from their nest. He did not use his epinephrine injector and lost consciousness shortly after developing generalized wheals.

We conclude that standard dosed single venom-based immunotherapy over 3 to 5 years effectively and long-lastingly protects the vast majority of Hymenoptera venom allergic patients.

### Treatment failure is related to a limited number of risk factors

Of a number of potential risk factors for treatment failure, only one reached statistical significance: severe anaphylaxis following the pre-VIT index sting was indicative of a higher risk of recurrence (*P* = 0.02) which is in accordance with the results of some previous studies [31,34], but was not supported by others [24,26,27,32,37]. Case series and epidemiological studies have clearly documented that both, the severity of anaphylaxis to the index sting as well as the risk of treatment failure, are related to an increase of the baseline serum tryptase concentration [38-41]. Tryptase determination is nowadays an integrative part of diagnostic assessment in Hymenoptera venom allergic patients, but was not routinely available during the first decade of our study. As a result, we could not retrospectively correlate the risk of treatment failure with baseline serum tryptase concentrations in our study group (tryptase concentrations were available in 87 patients and exceeding 11.4 μg/L in 9 cases).

Other potential risk factors including the patient’s age (*P* = 0.40), sex (*P* = 0.70), and preexisting atopy (*P* = 0.15), as well as the decline of venom specific IgE (*P* = 0.12) and skin test reactivity (*P* = 0.45) during VIT [24,25,27,31,42], the duration of treatment [24,34] (*P* = 0.56), the culprit insect [36] (*P* = 0.50), and repetitive exposure to Hymenoptera field stings (*P* = 0.07) did not significantly correlate with the outcome of VIT in our patient cohort. At least two aspects, however, require critical reflection: i) Higher relapse rates have been observed in honey bee venom allergic patients by other authors [36]. We assume that our inability to confirm a relation between the culprit stinging insect and the outcome of VIT may be due to a statistical type 2 error, meaning that the association would have proved statistically significant in a cohort of patients containing a higher proportion of honey bee venom allergic individuals. ii) Though there was a tendency towards an increased risk of relapse in individuals repeatedly suffering Hymenoptera re-stings during and after VIT (*P* = 0.07), we could not statistically confirm repetitive stings as a risk factor for treatment failure as reported in previous studies [23,24]. Our findings, however, demonstrate that tolerated field stings do not reliably predict long-term protection as illustrated by patients 2, 3, 10, 11, and 19 (Table 4) who did not react to Hymenoptera stings during VIT, but had anaphylactic reactions to re-stings following discontinuation of treatment.

### Most double sensitized patients are sufficiently protected by single VIT

In Northern Europe, the vast majority of sting-related anaphylactic reactions can be attributed to stings of honey bees and *Vespula* species, *Vespula* stings being the most common elicitor of venom-induced anaphylaxis in Germany. Our 55.3% rate of patients with double sensitization to both venoms is in line with previous studies reporting up to 59% of double positive sera [4], depending on the system used for determination of specific IgE [4,43]. Double positivity was more often detected serologically than in intradermal tests (Table 2) which is again in accordance with earlier findings [43]. As described previously [4,36], serological (*P* < 0.001) and intradermal test reactivity (*P* = 0.001) as well as the rate of double positivity (*P* < 0.001) were higher in bee venom allergic patients.

Other than venom “cross-reactivity” due to partial sequence identity of protein allergens or cross-reactive carbohydrate determinants, “independent” or major allergen-based double sensitization, has been claimed to necessitate VIT with both venoms [4,6,10,13]. A historical study published in 1992, however, suggested that single venom-based immunotherapy effectively protects most patients despite reactivity to more than one venom [44], but did not statistically compare the outcome of VIT in mono sensitized and double sensitized individuals.

In our allergy clinic, VIT is usually restricted to a single venom (see Methods section and Figure 1 for the process of decision making). During the study period, only 14 patients received double VIT with honey bee and *Vespula* venom and were therefore excluded from evaluation (no relapses were reported in this small group of patients). Our approach is based on the assumption that sensitization to one or several Hymenoptera venoms is a common finding in approximately 25% of an average adult population [15,45], reflecting previous exposure to Hymenoptera stings rather than clinical allergy. Though asymptomatic sensitization is associated with an intermediate risk of subsequent sting-related anaphylaxis (which may reach 17% [15,46]), it is international consensus that it is not an indication for VIT. In our opinion this also applies to patients with a history of anaphylactic sting reactions to one insect and a hitherto asymptomatic sensitization to another – regardless of its pattern or origin.

Our study specifically compared the efficacy of single venom-based immunotherapy in mono sensitized and double sensitized individuals. Relapse rates in both groups (6.0% in mono sensitized and 7.8% in double sensitized patients) were within or even below commonly reported recurrence rates of 10 to 15%. Still, there was a tendency (though not statistically significant) towards an increased risk of field sting-related anaphylaxis in double sensitized patients (*P* = 0.56). Of 28 patients suffering re-sting reactions, 5 double sensitized individuals reacted to an unidentified stinging insect (Table 4, patients 7, 8, 21, 25, and 26). One double sensitized patient (patient 5) treated with honey bee VIT subsequently reacted to a *Vespula* sting with a grade I reaction. This indicates a remaining risk of a wrong or insufficient choice of venom when using our above mentioned algorithm which is based on the patient’s ability to provide correct sting-related information or even visual identification of the culprit stinging insect. Study data, however, demonstrate that the future risk of anaphylactic reactions to *either* honey bee or yellow jacket stings was well below the reported 17% rate [15,46] of anaphylactic sting reactions in healthy adults with asymptomatic sensitization. While still not reaching statistical significance, there was a tendency towards an increased risk of relapsing sting-induced anaphylaxis in patients with equal reactivity to both venoms in diagnostic tests (*P* = 0.15) when compared to the total group of double sensitized patients (*P* = 0.56). We conclude that, in double sensitized individuals, the respective degree of skin test and serological reactivity to both venoms is indicative of their clinical relevance, meaning that the culprit insect venom is likely to produce a stronger response in diagnostic tests. It is self-evident that, since there is no absolute correlation between specific IgE levels or the degree of skin test reactivity with clinical responses to Hymenoptera stings [1], this conclusion is restricted to the intra-individual comparison of test results in double sensitized patients.

The overall low rate of relapsing anaphylaxis following Hymenoptera field stings (7.1%) and the exceedingly low rate of severe allergic reactions (0.5%) as observed in our patient cohort confirm effective long-term protection of mono sensitized and most double sensitized individuals by single VIT. We conclude that time-consuming and expensive double VIT should reasonably be reserved to clinically double allergic patients with a history of systemic reactions to both venoms. It may additionally be considered in double sensitive patients with equal reactivity to both venoms who have not reliably identified the responsible stinging insect. In cases of doubt, additional risk factors such as an elevated baseline serum tryptase, a history of particularly severe anaphylactic reactions to pre-VIT index stings, or a high degree of exposure to Hymenoptera stings should be taken into account. The recent introduction of assays determining specific IgE to recombinant major allergens rApi m1, rVes v5 and rVes v1 [4-6] might further help to identify double sensitized individuals at an increased risk of future anaphylactic reactions to their “non-culprit” insect. However, prospective clinical trials are needed to investigate the positive predictive value of major allergen-based IgE-sensitization.

### Conclusions and clinical implications

i) In addition to a comprehensive history, the respective degree of skin test and serological reactivity should be taken into account to identify the appropriate venom for immunotherapy in double sensitized patients.

ii) 3 to 5 years of standard-dosed single venom-based immunotherapy result in long-term effective protection of the vast majority of Hymenoptera venom allergic patients. Recurring anaphylaxis to Hymenoptera field-stings during or after VIT is generally less severe than the pre-VIT sting reaction.

iii) Treatment failure is related to a limited number of risk factors including a history of severe pre-VIT sting-induced anaphylaxis.

iv) No significant correlation between double sensitization and relapsing venom-induced anaphylaxis could be statistically confirmed in our study group, indicating that VIT can be reasonably confined to a single venom in most patients.

## Abbreviations

VIT: (Venom immunotherapy).

## Competing interests

The authors declare that they have no competing interest.

## Authors’ contribution

JS participated in the design of the study, collection and interpretation of data, and drafted the manuscript. BH participated in the design of the study, collected the data, and assisted in drafting the manuscript. JH performed the statistical analysis, and helped to draft the manuscript. AK participated in the collection of data, and critically revised the manuscript. AT participated in the design of the study, collection and interpretation of data, and critically revised the manuscript. All authors read and approved the final manuscript.

## References

[B1] Golden DB Moffitt J Nicklas RA Freeman T Graft DF Reisman RE Tracy JM Bernstein D Blessing-Moore J Cox L Stinging insect hypersensitivity: a practice parameter update 2011 J Allergy Clin Immunol 2011 127 852 854 e851-823 10.1016/j.jaci.2011.01.025 21458655

[B2] Krishna MT Ewan PW Diwakar L Durham SR Frew AJ Leech SC Nasser SM Diagnosis and management of hymenoptera venom allergy: British Society for Allergy and Clinical Immunology (BSACI) guidelines Clin Exp Allergy 2011 41 1201 1220 10.1111/j.1365-2222.2011.03788.x 21848758

[B3] Bonifazi F Jutel M Bilò BM Birnbaum J Müller U Prevention and treatment of hymenoptera venom allergy: guidelines for clinical practice Allergy 2005 60 1459 1470 10.1111/j.1398-9995.2005.00960.x 16266376

[B4] Müller UR Johansen N Petersen AB Fromberg-Nielsen J Haeberli G Hymenoptera venom allergy: analysis of double positivity to honey bee and Vespula venom by estimation of IgE antibodies to species-specific major allergens Api m1 and Ves v5 Allergy 2009 64 543 548 10.1111/j.1398-9995.2008.01794.x 19120073

[B5] Müller U Schmid-Grendelmeier P Hausmann O Helbling A IgE to recombinant allergens Api m 1, Ves v 1, and Ves v 5 distinguish double sensitization from crossreaction in venom allergy Allergy 2012 67 1069 1073 10.1111/j.1398-9995.2012.02847.x 22676144

[B6] Hofmann SC Pfender N Weckesser S Huss-Marp J Jakob T Added value of IgE detection to rApi m 1 and rVes v 5 in patients with Hymenoptera venom allergy J Allergy Clin Immunol 2011 127 265 267 10.1016/j.jaci.2010.06.042 20719373

[B7] Mittermann I Zidarn M Silar M Markovic-Housley Z Aberer W Korosec P Kosnik M Valenta R Recombinant allergen-based IgE testing to distinguish bee and wasp allergy J Allergy Clin Immunol 2010 125 1300 1307 e1303 10.1016/j.jaci.2010.03.017 20466415

[B8] Jappe U Raulf-Heimsoth M Hoffmann M Burow G Hübsch-Müller C Enk A In vitro hymenoptera venom allergy diagnosis: improved by screening for cross-reactive carbohydrate determinants and reciprocal inhibition Allergy 2006 61 1220 1229 10.1111/j.1398-9995.2006.01232.x 16942573

[B9] Hemmer W Focke M Kolarich D Wilson IB Altmann F Wohrl S Gotz M Jarisch R Antibody binding to venom carbohydrates is a frequent cause for double positivity to honeybee and yellow jacket venom in patients with stinging-insect allergy J Allergy Clin Immunol 2001 108 1045 1052 10.1067/mai.2001.120013 11742287

[B10] Straumann F Bucher C Wüthrich B Double sensitization to honeybee and wasp venom: immunotherapy with one or with both venoms? Value of FEIA inhibition for the identification of the cross-reacting ige antibodies in double-sensitized patients to honeybee and wasp venom Int Arch Allergy Immunol 2000 123 268 274 10.1159/000024453 11112864

[B11] Reisman RE Müller UR Wypych JI Lazell MI Studies of coexisting honeybee and vespid-venom sensitivity J Allergy Clin Immunol 1984 73 246 252 10.1016/S0091-6749(84)80015-9 6699307

[B12] Sturm GJ Böhm E Trummer M Weiglhofer I Heinemann A Aberer W The CD63 basophil activation test in Hymenoptera venom allergy: a prospective study Allergy 2004 59 1110 1117 10.1111/j.1398-9995.2004.00400.x 15355471

[B13] Eberlein B Krischan L Darsow U Ollert M Ring J Double positivity to bee and wasp venom: improved diagnostic procedure by recombinant allergen-based IgE testing and basophil activation test including data about cross-reactive carbohydrate determinants J Allergy Clin Immunol 2012 130 155 161 10.1016/j.jaci.2012.02.008 22421265

[B14] Mertens M Amler S Moerschbacher BM Brehler R Cross-reactive carbohydrate determinants strongly affect the results of the basophil activation test in hymenoptera-venom allergy Clin Exp Allergy 2010 40 1333 1345 10.1111/j.1365-2222.2010.03535.x 20545702

[B15] Golden DB Insect sting allergy and venom immunotherapy: a model and a mystery J Allergy Clin Immunol 2005 115 439 447 10.1016/j.jaci.2005.01.005 15753884

[B16] Muraro A Roberts G Clark A Eigenmann PA Halken S Lack G Moneret-Vautrin A Niggemann B Rance F The management of anaphylaxis in childhood: position paper of the European academy of allergology and clinical immunology Allergy 2007 62 857 871 10.1111/j.1398-9995.2007.01421.x 17590200

[B17] Stoevesandt J Hain J Kerstan A Trautmann A Over- and underestimated parameters in severe Hymenoptera venom-induced anaphylaxis: cardiovascular medication and absence of urticaria/angioedema J Allergy Clin Immunol 2012 130 698 704 10.1016/j.jaci.2012.03.024 22554708

[B18] Position paper Allergen standardization and skin tests. The European Academy of Allergology and Clinical Immunology Allergy 1993 48 48 82 8342740

[B19] Bilò BM Ruëff F Mosbech H Bonifazi F Oude-Elberink JN Diagnosis of Hymenoptera venom allergy Allergy 2005 60 1339 1349 10.1111/j.1398-9995.2005.00963.x 16197464

[B20] Boyle RJ Elremeli M Hockenhull J Cherry MG Bulsara MK Daniels M Oude Elberink JN Venom immunotherapy for preventing allergic reactions to insect stings Cochrane Database Syst Rev 2012 10 008838 10.1002/14651858.CD008838.pub2 PMC8734599 23076950

[B21] Golden DB Long-term outcome after venom immunotherapy Curr Opin Allergy Clin Immunol 2010 10 337 341 20610978 10.1097/ACI.0b013e32833bc0ba

[B22] Golden DB Kagey-Sobotka A Lichtenstein LM Survey of patients after discontinuing venom immunotherapy J Allergy Clin Immunol 2000 105 385 390 10.1016/S0091-6749(00)90092-7 10669863

[B23] Hafner T DuBuske L Kosnik M Long-term efficacy of venom immunotherapy Ann Allergy Asthma Immunol 2008 100 162 165 10.1016/S1081-1206(10)60425-5 18320918

[B24] Lerch E Müller UR Long-term protection after stopping venom immunotherapy: results of re-stings in 200 patients J Allergy Clin Immunol 1998 101 606 612 10.1016/S0091-6749(98)70167-8 9600496

[B25] Reisman RE Lantner R Further observations of stopping venom immunotherapy: comparison of patients stopped because of a fall in serum venom-specific IgE to insignificant levels with patients stopped prematurely by self-choice J Allergy Clin Immunol 1989 83 1049 1054 10.1016/0091-6749(89)90446-6 2732405

[B26] Golden DB Kwiterovich KA Kagey-Sobotka A Lichtenstein LM Discontinuing venom immunotherapy: extended observations J Allergy Clin Immunol 1998 101 298 305 10.1016/S0091-6749(98)70239-8 9525443

[B27] Golden DB Kwiterovich KA Kagey-Sobotka A Valentine MD Lichtenstein LM Discontinuing venom immunotherapy: outcome after five years J Allergy Clin Immunol 1996 97 579 587 10.1016/S0091-6749(96)70302-0 8621842

[B28] Oude Elberink JN de Monchy JG Kors JW van Doormaal JJ Dubois AE Fatal anaphylaxis after a yellow jacket sting, despite venom immunotherapy, in two patients with mastocytosis J Allergy Clin Immunol 1997 99 153 154 9003225 10.1016/s0091-6749(97)70314-2

[B29] Light WC Insect sting fatality 9 years after venom treatment (venom allergy, fatality) J Allergy Clin Immunol 2001 107 925 11344365 10.1067/mai.2001.114985

[B30] Golden DB Fatal insect allergy after discontinuation of venom immunotherapy J Allergy Clin Immunol 2001 107 925 926 11344366 10.1067/mai.2001.115095

[B31] Reisman RE Duration of venom immunotherapy: relationship to the severity of symptoms of initial insect sting anaphylaxis J Allergy Clin Immunol 1993 92 831 836 10.1016/0091-6749(93)90060-S 8258617

[B32] Müller U Berchtold E Helbling A Honeybee venom allergy: results of a sting challenge 1 year after stopping successful venom immunotherapy in 86 patients J Allergy Clin Immunol 1991 87 702 709 10.1016/0091-6749(91)90392-2 2005323

[B33] Haugaard L Norregaard OF Dahl R In-hospital sting challenge in insect venom-allergic patients after stopping venom immunotherapy J Allergy Clin Immunol 1991 87 699 702 10.1016/0091-6749(91)90391-Z 2005322

[B34] Keating MU Kagey-Sobotka A Hamilton RG Yunginger JW Clinical and immunologic follow-up of patients who stop venom immunotherapy J Allergy Clin Immunol 1991 88 339 348 10.1016/0091-6749(91)90095-6 1890261

[B35] van Halteren HK van der Linden PW Burgers JA Bartelink AK Discontinuation of yellow jacket venom immunotherapy: follow-up of 75 patients by means of deliberate sting challenge J Allergy Clin Immunol 1997 100 767 770 10.1016/S0091-6749(97)70271-9 9438484

[B36] Müller U Helbling A Berchtold E Immunotherapy with honeybee venom and yellow jacket venom is different regarding efficacy and safety J Allergy Clin Immunol 1992 89 529 535 10.1016/0091-6749(92)90319-W 1740583

[B37] Golden DB Kagey-Sobotka A Valentine MD Lichtenstein LM Dose dependence of Hymenoptera venom immunotherapy J Allergy Clin Immunol 1981 67 370 374 10.1016/0091-6749(81)90082-8 7229226

[B38] Haeberli G Bronnimann M Hunziker T Müller U Elevated basal serum tryptase and hymenoptera venom allergy: relation to severity of sting reactions and to safety and efficacy of venom immunotherapy Clin Exp Allergy 2003 33 1216 1220 10.1046/j.1365-2222.2003.01755.x 12956741

[B39] Ruëff F Przybilla B Bilò MB Müller U Scheipl F Aberer W Birnbaum J Bodzenta-Lukaszyk A Bonifazi F Bucher C Predictors of severe systemic anaphylactic reactions in patients with Hymenoptera venom allergy: importance of baseline serum tryptase-a study of the European Academy of Allergology and Clinical Immunology Interest Group on Insect Venom Hypersensitivity J Allergy Clin Immunol 2009 124 1047 1054 10.1016/j.jaci.2009.08.027 19895993

[B40] Niedoszytko M de Monchy J van Doormaal JJ Jassem E Oude Elberink JN Mastocytosis and insect venom allergy: diagnosis, safety and efficacy of venom immunotherapy Allergy 2009 64 1237 1245 10.1111/j.1398-9995.2009.02118.x 19627278

[B41] González De Olano D Alvarez-Twose I Esteban-López MI Sánchez-Muñoz L de Durana MD Vega A García-Montero A González-Mancebo E Belver T Herrero-Gil MD Safety and effectiveness of immunotherapy in patients with indolent systemic mastocytosis presenting with Hymenoptera venom anaphylaxis J Allergy Clin Immunol 2008 121 519 526 10.1016/j.jaci.2007.11.010 18177694

[B42] Randolph CC Reisman RE Evaluation of decline in serum venom-specific IgE as a criterion for stopping venom immunotherapy J Allergy Clin Immunol 1986 77 823 827 10.1016/0091-6749(86)90379-9 3711549

[B43] Egner W Ward C Brown DL Ewan PW The frequency and clinical significance of specific IgE to both wasp (Vespula) and honey-bee (Apis) venoms in the same patient Clin Exp Allergy 1998 28 26 34 9537776 10.1046/j.1365-2222.1998.00176.x

[B44] Reisman RE Livingston A Venom immunotherapy: 10 years of experience with administration of single venoms and 50 micrograms maintenance doses J Allergy Clin Immunol 1992 89 1189 1195 10.1016/0091-6749(92)90304-K 1607552

[B45] Golden DB Marsh DG Kagey-Sobotka A Freidhoff L Szklo M Valentine MD Lichtenstein LM Epidemiology of insect venom sensitivity JAMA 1989 262 240 244 10.1001/jama.1989.03430020082033 2739018

[B46] Golden DB Marsh DG Freidhoff LR Kwiterovich KA Addison B Kagey-Sobotka A Lichtenstein LM Natural history of Hymenoptera venom sensitivity in adults J Allergy Clin Immunol 1997 100 760 766 10.1016/S0091-6749(97)70270-7 9438483

